# Structural and Functional Changes in Mild Cognitive Impairment in Parkinson’s Disease

**DOI:** 10.3390/medicina60010033

**Published:** 2023-12-24

**Authors:** Halil Güllüoğlu, Duygu Hünerli, Raif Çakmur, Berril Dönmez Çolakoğlu, Emel Ada, Görsev Yener

**Affiliations:** 1 Department of Neurology, Izmir Ekonomi University Medical Point Hospital, Izmir 35080, Turkey; 2 Department of Neuroscience, Dokuz Eylul University Institute Health Sciences, Izmir 35210, Turkey; dhunerli@gmail.com; 3 Department of Neurology, Dokuz Eylul University School of Medicine, Izmir 35210, Turkey; raif.cakmur@deu.edu.tr (R.Ç.); berril.donmez@deu.edu.tr (B.D.Ç.); 4 Department of Radiology, Dokuz Eylul University School of Medicine, Izmir 35210, Turkey; emel.ada@deu.edu.tr; 5 Izmir Biomedicine and Genome Center, Izmir 35340, Turkey; gorsev.yener1@gmail.com; 6 Department of Neurolohy, Faculty of Medicine, Izmir University of Economics, Izmir 35330, Turkey

**Keywords:** cognitive impairment, Parkinson’s disease, dementia, neuropsychology testing

## Abstract

*Background and Objectives:* The pathophysiology of mild cognitive impairment in Parkinson’s disease (PD-MCI) is still not fully elucidated. It has been shown in a few studies in the literature that volume loss in the occipital, parietal and frontal cortices and atrophy in the hippocampus of PD-MCI patients can occur in the early stages of PD. The aim of this study was to evaluate the relationship between gray and white matter volumes and different neuropsychological tests and volumetric magnetic resonance imaging parameters in patients with mild cognitive impairment in Parkinson’s disease (PD-MCI). *Materials and Methods:* Twenty-six PD-MCI and twenty-six healthy elderly (HC) were included in this study. *Results:* We found that Mini Mental State Examination, Trail Making Test Part A, Clock Drawing Test, Benton Line Judgment Orientation Test and pentagon figure-copying scores were impaired in PD-MCI patients due to the decrease in brain volumes. *Conclusions:* Our study revealed that among PD-MCI patients, there was a more noticeable decline in White matter volume (WMV) based on volumetric Magnetic Resonance Imaging (MRI) compared to the localized loss of GMV. We think that these abnormal neuropsychological tests in PD-MCI patients can be used as pretests in the evaluation of the stage of transition to dementia.

## 1. Introduction

Cognitive impairment is one of the most common non-motor complications in Parkinson’s disease (PD) [1,2,3,4]. Cognitive impairment can occur at any stage of the disease prior to motor symptoms. While cognitive dysfunction is seen at a rate of 25% in the early stages of the disease, this rate can reach up to 80% in the late stages. Mild cognitive impairment frequently progresses to dementia [5,6,7].

The pathophysiology of mild cognitive impairment in PD (PD-MCI) is still not fully elucidated. It has been shown in a few studies in the literature that volume loss in the occipital, parietal and frontal cortices and atrophy in the hippocampus of PD-MCI patients can occur in the early stages of PD [1,2,4]. The aim of this study is to evaluate gray and white matter volumes in relation to different neuropsychological tests in PD-MCI patients with volumetric magnetic resonance imaging (MRI) parameters.

## 2. Materials and Methods

### 2.1. Participant Selection

In the present study, twenty-six individuals with PD-MCI (age 67.77 ± 7.44 (mean ± SD); 8 female and 18 male) and twenty-six healthy elderly participants (HC) (age 67.77 ± 7.48 (mean ± SD); 12 female and 14 male) were included. All individuals provided written informed consent prior to voluntary participation in the study, and the study protocol was approved by the local ethical committee.

Patients with PD were recruited from the Movement Disorders Outpatient Clinic in the Department of Neurology at Dokuz Eylül University Hospital. The diagnosis of PD was determined according to the UK Parkinson’s Disease Society Brain Bank criteria [8]. The severity of motor symptoms was evaluated via The Unified Parkinson’s Disease Rating Scale (UPDRS) Part III [9], and disease severity was assessed with the scale of Hoehn and Yahr [10]. The criteria for including PD-MCI patients were as follows: (1) a confirmed clinical diagnosis of idiopathic PD, (2) stable control of motor symptoms via dopaminergic treatment and (3) a Hoehn and Yahr stage of III or lower. The exclusion criteria for those patients with PD-MCI were as follows: (1) the presence of dementia [11]; (2) a history of or the presence of visual hallucinations; (3) a history of and/or the presence of psychiatric disorders and being a recipient of treatment with medications affecting cognition such as antidepressants, anxiolytics and antipsychotics; (4) patients with a history of drug-induced dopamine dysregulation; (5) a history of and/or the presence of vascular brain lesions and cortical atrophy; (6) severe tremors preventing assessments; and (7) treatment with deep brain stimulation, jejunal levodopa or subcutaneous apomorphine.

Levodopa equivalent daily doses (LEDs) were also calculated using a standardized formula for all dopamine replacement therapies received by the patients [12]. All individuals with PD-MCI were on the following anti-Parkinsonian treatment at the time of assessment: L-dopa monotherapy (*n* = 10), dopamine agonist monotherapy (*n* = 2), MAO-B inhibitor (*n* = 2) or combined treatment (*n* = 12). The neuropsychological and electrophysiological assessments of PD-MCI patients were performed during their “on” periods.

A further 26 healthy elderly participants were enrolled from various community sources via bulletin board announcements. The exclusion criteria for the healthy elderly group were as follows: (1) a history of or the presence of any neurological abnormalities and/or cognitive impairment (Mini Mental State Examination, MMSE, scoring ≥ 27); and (2) a history of psychiatric disorders, cerebral atrophy, vascular lesions, head trauma, seizures, strokes and alcohol and/or drug abuse misuse. Participants with depressive symptoms (scoring 14 > on the Yesavage Geriatric Depression Scale and GDS) were also excluded from all groups [13].

### 2.2. Neuropsychological Measures

Neuropsychological performance was evaluated by trained neuropsychologists. The neuropsychological tests were used for the diagnosis of MCI based on the criteria recommended by [14]. Accordingly, raw neuropsychological test scores were transformed into Z-scores based on normative data in order to generate cognitive domains. In doing so, our primary emphasis was solely on the visuospatial functions among PD-MCI patients.

Global cognitive status was assessed using the Mini Mental State Examination (MMSE [15]) and the Montreal Cognitive Assessment Scale (MoCA [16]). Visuospatial functions were assessed using the Trail Making Test Part A (TMT [17]), Benton Line Judgment Orientation Test (BLOT [18]), Clock Drawing Test [19] and the pentagon-drawing item in the MMSE. The demographic and clinical characteristics and the Z-scores of cognitive tests among healthy elderly and PD-MCI participants are shown in Table 1.

### 2.3. MRI Acquisition, Preprocessing and Analysis

MRI was acquired according to the ADNI protocol (www.adni.loni.usc.edu [accessed on 10 May 2023]). For each subject, a high-resolution T1-weighted volumetric MR scan was obtained at the Dokuz Eylül University Neuroradiology Unit, Izmir, Turkey, using the 1.5 Tesla Philips Achieva system, including coronal 3D T1-weighted TFE sequences (TR: 9 ms; TE: 4 ms; FOV: 240 mm; matrix: 256; slice thickness: 1 mm; NSA: 1). Gray matter volume measurements were performed with the CAT12 toolbox (Computational Anatomy Toolbox, http://dbm.neuro.uni-jena.de/cat/ [accessed on 12 May 2023]) using the MATLAB-based (Mathworks, Sherborn, MA, USA) SPM12 software (Statistical Parametric Mapping, http://www.fil.ion.ucl.ac.uk/spm/software/spm12 [accessed on 5 April 2023]).

Three-dimensional T1-weighted images were first converted from DICOM format to NIFTI format. Secondly, the starting points of the images were manually corrected so that the *x*, *y* and *z* coordinates of the anterior commissure corresponded to the 0,0,0 point. This was carried out in order to align the MRI images to the Montreal Neurological Institute (MNI)’s template. Thirdly, the segmentation process was carried out based on the parameters recommended in the CAT12 user manual.

As a result of the segmentation process, 3D T1-weighted images were separated into gray matter, white matter and cerebrospinal fluid. The CAT12 “Estimate Mean Values inside the Region of Interest (ROI)” function was applied using the LPBA40 (LONI Probabilistic Brain Atlas, 101) atlas in order to obtain mean volume values in different ROIs. The average volume values for each ROI were extracted separately.

Gray matter volumes were also normalized to eliminate differences due to the individuals’ head size. The normalization process was performed by multiplying each obtained volume value with the volumetric normalization coefficient automatically calculated by SIENAX (Structural Image Evaluation using Normalization of Atrophy Cross-Sectional) [20].

### 2.4. Statistical Analysis

The SPSS 25.0 (IBM Corporation, Armonk, NY, USA) and Medcalc 14 (Acacialaan 22, B-8400 Ostend, Belgium) programs were used to analyze variables. The conformity of the data to the normal distribution was evaluated with the Shapiro–Wilk Francia test, while the homogeneity of variance was evaluated with the Levene test. In the comparison of two independent groups according to quantitative variables, the Independent Sample T test was used together with Bootstrap results, while the Mann–Whitney U test was used together with Monte Carlo results. In the comparison of categorical variables, the Pearson chi-square and Fisher’s exact tests were tested with the Monte Carlo simulation technique. Sensitivity, specificity, positive predictivity and negative predictivity ratios for the relationship between the classification of the cut-off value were calculated according to the variables and the actual classification. They were analyzed and expressed via ROC (receiver operating curve) curve analysis. A logistic regression test was used with the Backward method to determine the cause–effect relationship between the categorical dependent group variable and the explanatory variables. While quantitative variables were expressed as a mean (standard deviation) and median (minimum–maximum) in the tables, categorical variables were shown as *n* (%). The variables were analyzed at a 95% confidence level, and a *p*-value of less than 0.05 was deemed significant.

The correlation coefficient between the variable Z-Trail Making Test, Part A (total duration in seconds to complete the task), which was significant in the ROC analysis, and the Trail Making Test, Part A (total duration in seconds to complete the task) variable, which was significant in ROC analysis, was 1. Therefore, only the Trail Making Test Part A (total duration in seconds to complete the task) variable was included in the logistic regression test. Likewise, the correlation coefficient between the Z-Benton Line Orientation Test’s total score variable and the Benton Line Orientation Test’s total score variable was 1. From this point of view, only the Benton Line Orientation Test’s total score variable was included in the logistic regression test, although it was significant in both variables.

## 3. Results

The demographic, clinical and neuropsychological assessment of HC and PD-MCI patients are shown in Table 1 and Table 2.

There were no significant differences among groups regarding age, gender, handedness and years of education. The PD-MCI group showed decreased MMSE, TMT Part A, Clock Drawing, BLOT, and pentagon figure-copying scores compared with those in the HC group. In the comparison of the PD-MCI group and the HC group, it was observed that there was a statistical decrease in MMSE, TMT Part A, Clock Drawing, BLOT, and pentagon figure-copying scores (Table 3).

**Figure 1:**
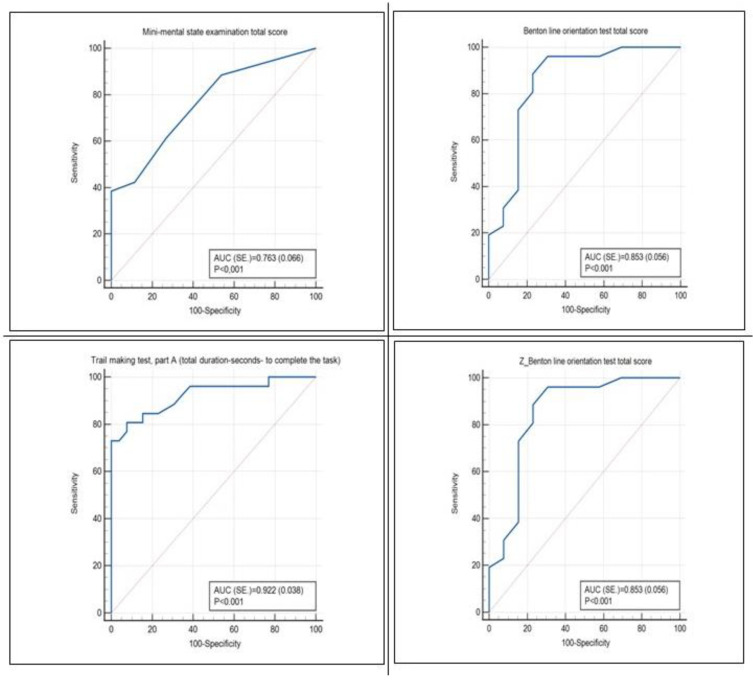

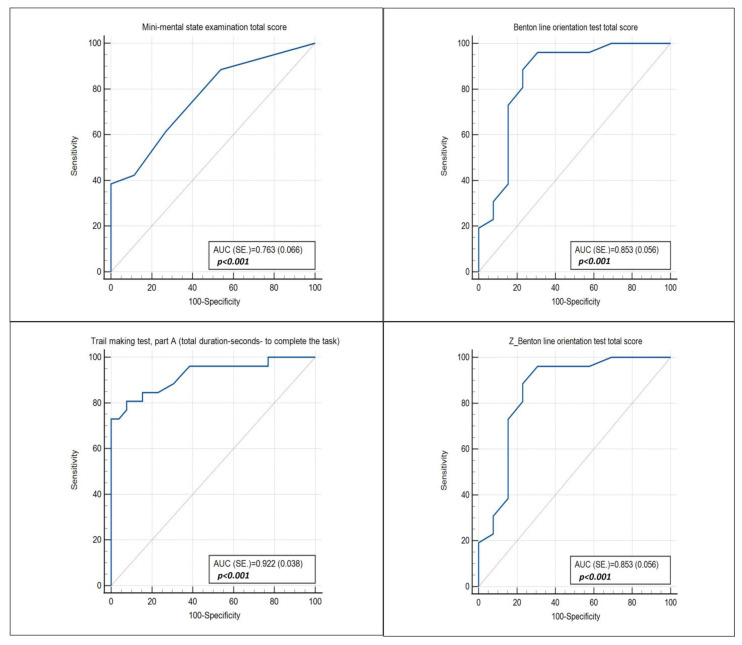
ROC curve analysis for diagnostic sensitivity analysis.

White matter volume (WMV) and total brain volume (TBV) were significantly lower in PD-MCI patients. Although there was no statistically significant difference between the two groups in terms of gray matter volume (GMV), the right angular gyrus, right superior occipital gyrus and left middle occipital gyrus volumes of PD-MCI patients were statistically significant. The PD-MCI group had a significantly reduced angular gyrus, superior occipital gyrus and middle occipital gyrus in terms of total volume compared to that in the HC group. There were no other significant subcortical GMV differences between PD-MCI patients and HC (Table 3, Table 4 and Table 5) (Figure 1).

## 4. Discussion

Although various studies have been conducted on the neuropathological basis of cognitive impairment and dementia in PD, there are still some unexplained points, especially in PD-MCI patients. Furthermore, we currently lack an initial supplementary examination for determining which individuals with PD-MCI will eventually develop dementia. Our research involved an exploration of the brain’s structural pathological changes and their impact on neuropsychological tests with respect to PD-MCI patients. Through this, we identified indicators that can serve as early signs in the progression of dementia. We observed abnormalities in both the neuropsychological assessments and volumetric MRI scans of our PD-MCI patients when compared to those from the HC group.

We investigated how MMSE, TMT Part A, Clock Drawing, BLOT, and pentagon figure-copying scores were negatively affected in PD-MCI patients, which we attributed to a reduction in brain volumes. Hence, we posit that certain neuropsychological tests, which we have identified as atypical in PD-MCI patients, could potentially serve as initial indicators for assessing the subsequent phase of cognitive decline, namely the transitional stage leading to dementia.

Neuropsychological tests can be used to identify patients whose cognitive functions are affected. Although the MMSE is the most commonly used neuropsychological test in the literature, it is not possible to talk about a homogeneous distribution regarding the use of the tests [3]. We performed the MMSE, MoCA, TMT Part A, Clock Drawing, BLOT, and pentagon figure-copying tests on our participants. We found that all tests were affected in PD-MCI patients. It is important to detect impairments in detailed neuropsychological evaluations in the early stages of PD-MCI in patients. Taking this perspective into consideration, we advocate for the periodic monitoring of these neuropsychological assessments in order to ascertain the progression to dementia during the transitional stage in PD-MCI patients. Many volumetric MRI studies have reported that GMV loss is evident in the temporal, parietal, frontal, occipital and limbic regions in PD-MCI and diffuse GMV loss in patients, who progress to the dementia phase [21,22,23,24,25]. It has been stated in previous studies that hippocampal volume loss is one of the most important factors predicting the development of mild cognitive impairment and dementia in Parkinson’s disease [26,27,28,29]. It has been stated that hyperintensities in white matter affect the longitudinal cognitive status in PD [26,30,31].

According to Mihaescu et al. [32], PD-MCI encompasses various subtypes, suggesting that categorizing this mild cognitive impairment within PD subtypes could lead to improved prognostic accuracy. According to the authors, structural and functional changes occur at different rates and in different brain regions, and progressive cognitive impairment occurs with increasing gray matter loss. Guo et al. emphasized that age is a risk factor for cognitive decline in Parkinson’s patients. They indicated that the decrease in information transmission efficiency of the cerebellum thalamus–cortex loop contributes to this situation [33]. While it is widely recognized that PD-MCI patients exhibit neuropsychological test impairments linked to GMV reduction, to the best of our knowledge, there is no existing study in the literature that focuses on WMV reduction independently of GMV reduction, all while assessing the corresponding impact on neuropsychological tests. Our study is the first study in the literature in this respect. It was shown that the basis of TBV loss occurs with the loss of WMV without GMV. However, volume losses in the right angular gyrus, right superior occipital gyrus and left middle occipital gyrus were detected in PD-MCI patients. This finding means that in PD-MCI patients, the regional loss of GMV occurs at the earliest disease stages. In this case, it shows us that especially in the early stages of the disease, volumetric MRI should be performed even if there is no cognitive impairment in patients, since it can identify neurodegeneration in vivo.

Our study revealed that among PD-MCI patients, there was a more noticeable decline in WMV based on volumetric MRI compared to the localized loss of GMV. This leads us to propose that the cognitive manifestations of PD-MCI linked to white matter changes (atrophy) might serve as early indicators for monitoring dementia progression. Given the relatively limited sample size and confinement to a single tertiary institution in our study, conducting a larger-scale, multicenter investigation could potentially yield more precise and comprehensive outcomes.

## Figures and Tables

**Table 1:** Patient characteristics.

		Total	HC	PDMCI	*p*
		(*n* = 52)	(*n* = 26)	(*n* = 26)	
Age, mean (SD.)		67.77 (7.38)	67.77 (7.48)	67.77 (7.44)	0.999 ᵗ
Gender (female), *n* (%)		20 (38.5)	12 (46.2)	8 (30.8)	0.393 ᶜ
Education, median (min/max)		11 (2/15)	12 (5/15)	9 (2/15)	0.155 ᵘ
Hand dominance (right), *n* (%)		51 (98.1)	26 (100)	25 (96.2)	0.999 ᶠ
PDMCI domain (nonamnestic), *n* (%)		2 (7.7)	0 (0)	2 (7.7)	-
Years, median (min/max)		3 (1/25)	-	3 (1/25)	-
Hoehn Yahr, median (min/max)		2 (1.5/5)	-	2 (1.5/5)	-
UPDRS motor, median (min/max)		24 (3/40)	-	24 (3/40)	-
Levodopa equivalent daily dose (mg), median (min/max)		500 (100/1257)	-	500 (100/1257)	-
PD meds, *n* (%)					-
	L-dopa	10 (38.5)	0 (0)	10 (38.5)	
	Dopamine agonist	2 (7.7)	0 (0)	2 (7.7)	
	MAO-B	2 (7.7)	0 (0)	2 (7.7)	
	Combined	12 (46.2)	0 (0)	12 (46.2)	

ᵗ Independent two sample *t*-test (Bootstrap); ᶜ chi-square test (Monte Carlo); ᶠ Fisher’s exact test (Monte Carlo); ᵘ Mann–Whitney U test (Monte Carlo); SD. = standard deviation; min = minimum; max = maximum.

**Table 2:** Neuropsychological assessment of HC and PD-MCI patients.

	Total	HC	PD-MCI	*p*
	(*n* = 52)	(*n* = 26)	(*n* = 26)	
Montreal Cognitive Assessment total score, median (min/max)	23 (9/29)	−	23 (9/29)	-
MMSE-pentagon figure, *n* (%)	44 (84.6)	25 (96.2)	19 (73.1)	0.049 ᶠ
Yesavage Geriatric Depression Scale total score, median (min/max)	5 (0/13)	5 (0/13)	6 (0/13)	0.512 ᵘ
Mini Mental State Examination total score, median (min/max)	29 (19/30)	29 (27/30)	28 (19/30)	<0.001 ᵘ
Trail Making Test, Part A (total duration—second—to complete the task), mean (SD.)	73.73 (34.23)	51.28 (11.04)	96.18 (34.93)	0.001 ᵗ
Benton Line Orientation Test total score, median (min/max)	19 (11/30)	22.26 (13/30)	16.22 (11/23)	<0.001 ᵘ
Clock Drawing total score, median (min/max)	10 (0/10)	10 (8/10)	10 (0/10)	0.016 ᵘ
Z_Trail Making Test, Part A (total duration— seconds—to complete the task), mean (SD.)	2.03 (3.1)	0 (1)	4.07 (3.16)	0.001 ᵗ
Z_Benton Line Orientation Test total score, median (min/max)	−0.74 (−2.55/1.75)	0 (−2.1/1.75)	−1.37 (−2.55/0.17)	<0.001 ᵘ
Z_MMSE-pentagon figure, median (min/max)	−0.57 (−4.8/0.2)	0.01 (−4.8/0.2)	−1 (−4.8/0.2)	0.048 ᵘ
Z_Clock Drawing total score, median (min/max)	0.28 (−22.98/0.28)	0.01 (−4.37/0.28)	−1.5 (−22.98/0.28)	0.016 ᵘ
Visuospatial skills, median (min/max)	0.09 (−6.23/1.25)	0.1 (−1.28/0.61)	0.08 (−6.23/1.25)	0.768 ᵘ
Gray matter volume, mean (SD.)	41.63 (2.72)	42.08 (2.36)	41.18 (3.02)	0.249 ᵗ
White matter volume, mean (SD.)	35.82 (2.65)	36.62 (2.4)	35.01 (2.69)	0.030 ᵗ
Total brain volume, mean (SD.)	77.45 (4.01)	78.7 (3.12)	76.19 (4.45)	0.028 ᵗ

ᵗ Independent two sample *t*-test (Bootstrap); ᶠ Fisher’s exact test (Monte Carlo); ᵘ Mann–Whitney U test (Monte Carlo); SD. = standard deviation; min= minimum; max= maximum.

**Table 3:** Subcortical GMV differences between PD-MCI patients.

	Total	HC	PDMCI	*p*	Total	HC	PD-MCI	*p*
	(*n* = 52)	(*n* = 26)	(*n* = 26)		(*n* = 52)	(*n* = 26)	(*n* = 26)	
	Left				Right			
	Mean (SD.) or Median (Min/Max)				Mean (SD.) or Median (Min/Max)			
Superior Frontal Gyrus	1.9 (0.14)	1.9 (0.12)	1.91 (0.16)	0.985 ᵗ	1.86(1.53/2.19)	1.85(1.73/2.16)	1.88(1.53/2.19)	0.768 ᵘ
Middle Frontal Gyrus	1.41 (0.13)	1.41 (0.11)	1.42 (0.15)	0.797 ᵗ	1.44 (0.12)	1.44 (0.11)	1.43 (0.13)	0.765 ᵗ
Inferior Frontal Gyrus	0.66 (0.06)	0.66 (0.06)	0.66 (0.07)	0.651 ᵗ	0.72 (0.07)	0.71 (0.06)	0.72 (0.08)	0.974 ᵗ
Precentral Gyrus	0.74 (0.08)	0.75 (0.07)	0.73 (0.09)	0.281 ᵗ	0.73 (0.07)	0.73 (0.07)	0.72 (0.08)	0.714 ᵗ
Middle Orbitofrontal Gyrus	0.34 (0.03)	0.34 (0.03)	0.34 (0.04)	0.894 ᵗ	0.35 (0.03)	0.35 (0.02)	0.35 (0.04)	0.756 ᵗ
Lateral Orbitofrontal Gyrus	0.23 (0.02)	0.23 (0.02)	0.23 (0.03)	0.876 ᵗ	0.2 (0.02)	0.2 (0.01)	0.19 (0.03)	0.272 ᵗ
Gyrus Rectus	0.13 (0.01)	0.13 (0.01)	0.13 (0.01)	0.516 ᵗ	0.13 (0.01)	0.13 (0.01)	0.13 (0.01)	0.908 ᵗ
Postcentral Gyrus	0.62 (0.07)	0.63 (0.07)	0.61 (0.08)	0.272 ᵗ	0.58 (0.07)	0.59 (0.06)	0.58 (0.07)	0.644 ᵗ
Superior Parietal Gyrus	0.77 (0.07)	0.76 (0.07)	0.78 (0.06)	0.512 ᵗ	0.76 (0.08)	0.76 (0.08)	0.77 (0.09)	0.892 ᵗ
Supramarginal Gyrus	0.48 (0.05)	0.49 (0.04)	0.47 (0.06)	0.135 ᵗ	0.47 (0.05)	0.48 (0.05)	0.47 (0.06)	0.450 ᵗ
Angular Gyrus	0.63 (0.07)	0.64 (0.06)	0.61 (0.07)	0.122 ᵗ	0.68 (0.06)	0.7 (0.06)	0.67 (0.06)	0.050 ᵗ
Precuneus	0.44 (0.04)	0.44 (0.04)	0.44 (0.05)	0.704 ᵗ	0.44 (0.05)	0.44 (0.05)	0.44 (0.05)	0.691 ᵗ
Superior Occipital Gyrus	0.23 (0.03)	0.24 (0.02)	0.23 (0.03)	0.178 ᵗ	0.26 (0.03)	0.27 (0.03)	0.25 (0.03)	0.041 ᵗ
Middle Occipital Gyrus	0.75 (0.07)	0.77 (0.07)	0.72 (0.06)	0.029 ᵗ	0.77 (0.08)	0.79 (0.07)	0.76 (0.08)	0.159 ᵗ
Inferior Occipital Gyrus	0.41(0.33/0.49)	0.41(0.34/0.47)	0.38(0.33/0.49)	0.091 ᵘ	0.41 (0.04)	0.41 (0.03)	0.41 (0.04)	0.943 ᵗ
Cuneus	0.21 (0.02)	0.21 (0.02)	0.21 (0.03)	0.844 ᵗ	0.23(0.16/0.26)	0.23(0.16/0.26)	0.22(0.17/0.26)	0.406 ᵘ
Superior Temporal Gyrus	1.06 (0.09)	1.09 (0.09)	1.04 (0.1)	0.074 ᵗ	0.99 (0.1)	1.02 (0.09)	0.97 (0.11)	0.104 ᵗ
Middle Temporal Gyrus	0.9 (0.09)	0.91 (0.09)	0.89 (0.09)	0.290 ᵗ	0.94 (0.1)	0.96 (0.1)	0.93 (0.09)	0.152 ᵗ
Inferior Temporal Gyrus	0.84 (0.08)	0.85 (0.07)	0.83 (0.08)	0.285 ᵗ	0.9 (0.09)	0.91 (0.08)	0.89 (0.09)	0.586 ᵗ
Parahippocampal Gyrus	0.25 (0.02)	0.25 (0.02)	0.25 (0.02)	0.986 ᵗ	0.26 (0.02)	0.26 (0.02)	0.26 (0.02)	0.946 ᵗ
Lingual Gyrus	0.48 (0.05)	0.49 (0.05)	0.47 (0.05)	0.145 ᵗ	0.49 (0.05)	0.5 (0.04)	0.48 (0.05)	0.089 ᵗ
Fusiform Gyrus	0.54 (0.05)	0.54 (0.05)	0.53 (0.05)	0.331 ᵗ	0.53 (0.05)	0.53 (0.05)	0.52 (0.05)	0.493 ᵗ
Insula	0.37 (0.03)	0.37 (0.03)	0.37 (0.04)	0.769 ᵗ	0.37(0.29/0.42)	0.37(0.29/0.42)	0.36(0.3/0.41)	0.704 ᵘ
Cingulate Gyrus	0.52 (0.05)	0.52 (0.04)	0.51 (0.06)	0.252 ᵗ	0.58 (0.05)	0.59 (0.05)	0.58 (0.06)	0.416 ᵗ
Caudate	0.16 (0.03)	0.16 (0.03)	0.15 (0.02)	0.194 ᵗ	0.15 (0.02)	0.16 (0.02)	0.15 (0.02)	0.118 ᵗ
Putamen	0.23 (0.03)	0.23 (0.03)	0.23 (0.02)	0.258 ᵗ	0.23 (0.02)	0.24 (0.03)	0.22 (0.02)	0.088 ᵗ
Hippocampus	0.23 (0.03)	0.23 (0.02)	0.22 (0.03)	0.150 ᵗ	0.24 (0.03)	0.24 (0.03)	0.23 (0.03)	0.217 ᵗ

ᵗ Independent two sample *t*-test (Bootstrap); ᵘ Mann–Whitney U test (Monte Carlo); SD. = standard deviation; min = minimum; max = maximum.

**Table 4:** Sensitivity analysis of PD-MCI.

Reference: PD-MCI	Cut Off	Sensitivity	Specificity	+PV	−PV	AUC ± SE.	*p* Value
Mini Mental State Examination total score	≤26	38.46	100.00	100.0	61.9	0.763 ± 0.066	<0.001
Trail Making Test, Part A (total duration—seconds—to complete the task)	>65	80.77	92.31	91.3	82.8	0.922 ± 0.038	<0.001
Benton Line Orientation Test total score	≤20	88.46	76.92	79.3	87.0	0.853 ± 0.056	<0.001
Clock Drawing total score	≤8	30.77	96.15	88.9	58.1	0.626 ± 0.078	0.108
Z_Trail Making Test, Part A (total duration—seconds—to complete the task)	>1.24	80.77	92.31	91.3	82.8	0.922 ± 0.038	<0.001
Z_Benton Line Orientation Test total score	≤−0.05	88.46	76.92	79.3	87.0	0.853 ± 0.056	<0.001
Z_MMSE-pentagon Figure	≤−4.8	26.92	96.15	87.5	56.8	0.615 ± 0.079	0.142
Z_Clock Drawing total score	≤−4.37	30.77	96.15	88.9	58.1	0.626 ± 0.078	0.108
White matter volume	≤36.39	80.77	57.69	65.6	75.0	0.694 ± 0.074	0.009
Total brain volume	≤74.02	38.46	96.15	90.9	61.0	0.666 ± 0.076	0.030
Right angular gyrus	≤0.64	46.15	84.62	75.0	61.1	0.661 ± 0.076	0.034
Right superior occipital gyrus	≤0.25	57.69	76.92	71.4	64.5	0.651 ± 0.077	0.049
Left middle occipital gyrus	≤0.69	38.46	92.31	83.3	60.0	0.683 ± 0.074	0.013
Left inferior occipital gyrus	≤0.38	46.15	92.31	85.7	63.2	0.639 ± 0.080	0.083
Left superior temporal gyrus	≤1	46.15	84.62	75.0	61.1	0.649 ± 0.077	0.053
Right lingual gyrus	≤0.49	69.23	61.54	64.3	66.7	0.633 ± 0.078	0.089
Right putamen	≤0.24	84.62	42.31	59.5	73.3	0.633 ± 0.078	0.088

ROC (receiver operating curve) analysis (Honley and Mc Nell–Youden index J); AUC = area under the ROC curve; SE. = standard error; +PV = positive predictive value; −PV = negative predictive value.

**Table 5:** Multiple logistic regression of PD-MCI.

Dependent Reference Group: (PD-MCI)	Trail Making Test, Part A (Total Duration in Seconds to Complete the Task), Adjusted			
	Odds Ratio	95% C.I. for Odds Ratio		*p*
		Lower	Upper	
Mini Mental State Examination total score (≤26)	5.5	0.9	35.0	0.069
Trail Making Test, Part A (total duration—seconds—to complete the task) (>65)	92.2	5.5	1535.0	0.002
Benton Line Orientation Test total score (≤20)	13.4	1.9	96.4	0.010
White matter volume (≤36.39)	13.5	1.1	169.5	0.044
Total brain volume (≤74.02)	30.2	1.8	503.3	0.018
Predicted ratio	**Cut point**	**PDMCI**	**HC**	**All**
	0.5	88.5	92.3	90.4

Multiple logistic regression (method = backward stepwise (Wald)).

## Data Availability

All data generated for this analysis were from an anonymized database. The code for the analyses will be made available upon request.
